# TCRαβ^+^NK1.1^-^CD4^-^CD8^-^ double-negative T cells inhibit central and peripheral inflammation and ameliorate ischemic stroke in mice

**DOI:** 10.7150/thno.80307

**Published:** 2023-01-10

**Authors:** Dan Tian, Yuhualei Pan, Yushang Zhao, Huan Wang, Yue Tian, Lu Yang, Wen Shi, Chengjie Zhang, Yanbing Zhu, Yongbo Zhang, Songlin Wang, Dong Zhang

**Affiliations:** 1 General Surgery Department, Beijing Friendship Hospital, Capital Medical University, Beijing, China.; 2 Immunology Research Center for Oral and Systemic Health, Beijing Friendship Hospital, Capital Medical University, Beijing, China.; 3 Beijing Key Laboratory of Tolerance Induction and Organ Protection in Transplantation, Beijing, China.; 4 National Clinical Research Center for Digestive Diseases, Beijing, China.; 5 Beijing Laboratory of Oral Health, Capital Medical University School of Stomatology, Beijing, China.; 6 Beijing Clinical Research Institute, Beijing, China.; 7 Department of Neurology, Beijing Friendship Hospital, Capital Medical University, Beijing, China.; 8 Beijing Institute of Brain Disorders, Capital Medical University, Beijing, China.

**Keywords:** Ischemic stroke, Double-negative T cell, Regulatory T cell, Myeloid cell

## Abstract

**Background:** Excessive immune activation leads to secondary injury and impedes injured brain recovery after ischemic stroke. However, few effective methods are currently used for equilibrating immune balance. CD3^+^NK1.1^-^TCRβ^+^CD4^-^CD8^-^ double-negative T (DNT) cells which do not express NK cell surface markers are unique regulatory cells that maintain homeostasis in several immune-related diseases. However, the therapeutic potential and regulatory mechanism of DNT cells in ischemic stroke are still unknown.

**Methods:** Mouse ischemic stroke is induced by occlusion of the distal branches of the middle cerebral artery (dMCAO). DNT cells were adoptively transferred intravenously into ischemic stroke mice. Neural recovery was evaluated by TTC staining and behavioral analysis. Using immunofluorescence, flow cytometry, and RNA sequencing, the immune regulatory function of DNT cells was investigated at different time points post ischemic stroke.

**Results:** Adoptive transfer of DNT cells significantly reduces infarct volume and improves sensorimotor function after ischemic stroke. DNT cells suppress peripheral Trem1^+^ myeloid cell differentiation during the acute phase. Furthermore, they infiltrate the ischemic tissue via CCR5 and equilibrate the local immune balance during the subacute phase. During the chronic phase, DNT cells enhance Treg cell recruitment through CCL5, eventually developing an immune homeostatic milieu for neuronal recovery.

**Conclusions:** DNT cell treatment renders the comprehensive anti-inflammatory roles in specific phases of ischemic stroke. Our study suggests that the adoptive transfer of regulatory DNT cells may be a potential cell-based therapy for ischemic stroke.

## Introduction

Ischemic stroke is a leading cause of mortality and disability worldwide, and the economic costs of treatment and post-stroke care are substantial 1. Ischemic stroke elicits a strong neuroinflammatory response, and neuroinflammation is involved in all stages of the ischemic cascade from the early damaging events triggered by arterial occlusion to the late regenerative processes underlying post-ischemic tissue repair 2.

An ischemic event releases damage-associated molecular patterns (DAMPs) into systemic circulation. These DAMPs activate and recruit peripheral and intestinal immune cells to the infarct regions 3. Excessive immune activation causes secondary injury and impedes injured brain recovery after ischemic stroke 4. The infiltrating myeloid and lymphoid cells trigger or dampen inflammation in different phases through various mechanisms 5. Peripheral triggering receptor expressed on myeloid cells-1 (Trem1) activation in myeloid cells amplifies proinflammatory responses in the ischemic brain during the early stages 6. In contrast, regulatory T cells (Treg cells) docked at the injured vessel suppress neurotoxic astrogliosis and potentiate neurological recovery during the chronic phase 7, 8. Thus, rectifying the immune homeostasis to favor tissue repair is a potential therapeutic approach for ischemic stroke.

Accumulating evidence supports the multifaceted roles of T cell subsets in response to ischemic stroke 9. CD3^+^TCRαβ^+^CD4^-^CD8^-^ double-negative T (DNT) cells which do not express NK cell surface markers represent 1-3% total T cells in the peripheral blood and lymphoid organs, but they are crucial for maintaining immune homeostasis in various diseases. Our previous studies have demonstrated that DNT cells suppress T and B cell proliferation and function via perforin and granzyme B 10, 11. Moreover, the adoptive transfer of DNT cells can prevent immune-related diseases, such as type 1 diabetes, allergic asthma, and non-alcoholic steatohepatitis 12-14. Since ischemic stroke induces systemic and local secondary inflammation and regulatory DNT cells show promising anti-inflammatory functions, we examined the therapeutic effects of DNT cells in ischemic stroke.

In this study, *ex vivo* generated CD3^+^TCRαβ^+^NK1.1^-^CD4^-^CD8^-^ DNT cells were adoptively transferred to ischemic stroke mice to identify their therapeutic effects and immunological mechanisms. Our results revealed that DNT cell therapy promotes functional recovery and neural repair after ischemic stroke. DNT cells suppress peripheral Trem1 positive macrophage differentiation in the acute stage. Furthermore, DNT cells infiltrated the ischemic areas via C-C Chemokine Receptor 5 (CCR5) and equilibrated the local immune balance. Finally, DNT cells contribute to Treg cells recruitment into the ischemic areas through C-C Motif Chemokine Ligand 5 (CCL5) secretion and promote further neural recovery during the chronic stages after stroke. These findings provide clues for a therapeutic approach to stroke via regulatory DNT cells.

## Methods

### Mice

*Foxp3*-eGFP (B6.Cg-*Foxp3*^tm2(EGFP)Tch^/J), *Ccr5*^-/-^ (B6.129P2-*Ccr5*^tm1Kuz^/J), B6-GFP (C57BL/6-Tg(UBC-GFP)30Scha/J), B6 CD45.1 (B6.SJL-*Ptprc*^a^ *Pepc*^b^/BoyJ), and wild-type C57BL/6J mice were purchased from the Jackson Laboratory (ME, USA). The animals were housed and bred under specific pathogen-free conditions in a temperature-controlled environment under 12 h light/dark cycles at Beijing Friendship Hospital. Potential confounders such as the order of treatments and measurements, or animal/cage location were not controlled. All mice were randomised to the sham, dMCAO or treatment group (n ≥ 3 mice/group) according to a computer-generated block randomisation list without stratification. All experimental studies were approved with assurance identification numbers 20-2008 by the Institutional Animal Care and Use Committee (IACUC) of Beijing Friendship Hospital.

### *In vitro* generation and adoptive transfer of DNT cells

The DNT cells were converted and amplified *in vitro* as previously described 10, 12. Mature dendritic cells (mDCs) were harvested from lipopolysaccharide-stimulated bone marrow cells derived from C57BL/6J mice and separated according to CD86-positive selection. CD4^+^ T cells were negatively isolated using anti-mouse Ter119/TCRγδ/CD11b/NK1.1/CD8/Gr-1-PE negative magnetic isolation (Miltenyi) and incubated with C57BL/6J mDCs and 50 ng/mL rmIL-2 (PeproTech, USA) for 7 d. CD3^+^TCRβ^+^CD4^-^CD8^-^NK1.1^-^ DNT cells were sorted using a FACSAria II sorter (BD Biosciences, USA). DNT cells (5×10^6^) were adoptively transferred intravenously into ischemic stroke mice after 30 min or 2 h.

### Induction of focal cortical ischemic stroke in mice

Focal cerebral ischemic stroke targeting the right sensorimotor cortex, mainly involving the barrel cortex, is induced by occlusion of the distal branches of the middle cerebral artery (dMCAO). The dMCAO model of focal cerebral ischemia targeting the right sensorimotor cortex in mice was established according to previous publications 15, 16. Briefly, Anesthesia was induced with 2% isoflurane (RWD Life Science, China) and maintained during surgery using 1.5% isoflurane supplemented with regular air. Focal cerebral ischemic stroke was achieved by permanently occluding the distal branches of the right middle cerebral artery (dMCA) and a 7-min ligation of both common carotid arteries. Sham surgery was performed in the same manner as stroke surgery, except for occlusion. The animals were placed in the surgery room before double-blind surgery for at least 30 min for adaptation to the environment. The animals' body temperature was maintained at 37±0.5 °C during the surgery and for up to 2 h post-surgery. After anesthetic resuscitation, the animal was returned to the feeding room.

### 2,3,5-Triphenyl tetrazolium chloride (TTC) staining

Three days after the onset of dMCAO, animals in the different groups were sacrificed for the assessment of brain infarction volume. The brain was removed from the skull, placed in a brain matrix, and sliced into 1 mm coronal sections. The slices were incubated in 2% TTC (Sigma-Aldrich, USA) solution at 37 °C for 10 min and then stored in 4% paraformaldehyde for 24 h to visualize later. The caudal aspect of each slice was digitally imaged using a flatbed scanner. The infarct, ipsilateral hemisphere, and contralateral hemisphere areas were measured using the ImageJ software (version 1.8.0). The indirect method (subtracting the residual right hemisphere cortical volume from the intact left hemisphere cortical volume) was used to calculate the infarct volume. Infarct measurements were performed under double blind conditions.

### Behavioral analysis

The adhesive-removal test is a sensitive method for monitoring the severity and recovery of sensorimotor deficits after cerebral focal ischemia in mice. A small and modest adhesive tape was fixed to the forepaws of each mouse. The time required for the mouse to contact and remove the adhesive was recorded. The animals were trained to perform the task for 3 d prior to stroke induction. Animals that indicated an inability to accomplish the task were ommitted from formal experiments. Prior to stroke induction, we performed the adhesive-removal task to establish baseline performance, and then 1, 3, 7, 14, and 21 d post-stroke. The mean time required to contact and remove the stimuli from each paw was recorded.

For the whisker-evoked forelimb placing test, the mice were gently held by their torsos, and brushed their whiskers against the corner of a platform to elicit same-side forelimb being placed with both sides of the face. For the whisker-evoked cross-midline placing test, the mice were held by their torsos and rotated 45 degrees. The lower side of the whiskers was then brushed perpendicularly against the surface edge of the corner platform to elicit the opposite side (upper side) forelimb placed on the platform. The eyes of the mice were covered to avoid the effects of visual information on performance during the test. The trial was recorded as successful if the tested forelimb was placed on the platform after the whisker touch. The trial was recorded as a failure if the mouse was motionless or shook, but failed to place a limb on the platform. Trials in which animals struggled while being held were not counted. Each mouse was trained for ten trials on both sides for three days for habituation to the test before stroke-inducing. The protocol was performed 1, 3, 7, 14, and 21 d after the stroke. The experimenters were blinded to the grouping of the mice during all the behavioral tests.

### Isolation of single cells from brain tissues

The infarct lesions of brain tissue were harvested using fine forceps and digested [0.1 mg/L collagenase IV, 1% calf serum in phosphate-buffered saline (PBS)] at 37 °C for 40 min. Single-cell suspensions were filtered using a 70 μm cell strainer and washed with MACS buffer (5 g/L bovine serum albumin, 2 mM EDTA, PBS), which was used for subsequent experiments.

### Flow cytometry

Flow cytometry was performed with a BD Aria II and analyzed using FlowJo software (Tree Star Inc.). FITC anti-CD15 (MC-480), APC anti-CD133 (315-2C11), FITC anti-mouse CD45 (I3/2.3), APC anti-CD45.1 (A20), APC anti-CD3 (17A2), Pacific Blue anti-CD4 (GK1.5), APC/Cyanine7 anti-CD8 (53-6.7), PE/Dazzle 594 anti-NK1.1 (PK136), APC/Cyanine7 anti-Ly-6G (1A8), Brilliant Violet 605 anti-CD11b (M1/70), Brilliant Violet 421 anti-F4/80 (BM8), Brilliant Violet 421 anti-mouse LAP (TGF-β1) (TW7-16B4), PE anti-TNF-α (MP6-XT22), PE anti-mouse CD195 (CCR5) (HM-CCR5), PE anti-CCL5 (RANTES) (2E9/CCL5), PE/Cy7 anti-CD24 (M1/69) monoclonal antibodies and Zombie Aqua™ Fixable Viability Kit, PE Annexin V, and 7-AAD reagents were purchased from Biolegend (USA). PE anti-TREM-1 (174031) and APC anti-TREM-2 (237920) antibodies were purchased from R&D Systems (USA). Anti-Glast PE (ACSA-1) was purchased from Miltenyi (Germany). Gating strategies of neural progenitor and immune cell are shown in Figure S1A-C.

### RNA sequencing and informatics analysis

Infarct areas and peripheral blood mononuclear cells (PBMC) were isolated from ischemic stroke mice with or without DNT cell therapy. RNAs was obtained using an RNeasy Plus Micro Kit (Qiagen, Germany) and sequenced using a standard Illumina protocol (Annoroad Gene Technology, Beijing). The reads were mapped to the mouse genome (Mm9) using HISAT2. Gene counts were estimated using HTSeq. The online platform xCell (xcell.ucsf.edu) was used to enumerate immune subsets with RPKM (reads per kilobase million) from RNA sequencing. Principal component analysis (PCA) and differential expression were assessed using DESeq2 (version 1.34.0) 17. The R package clusterprofiler (4.2.2) was used to perform gene ontology (GO) enrichment of differentially expressed genes (DEGs) and gene set enrichment analysis (GSEA) of all genes 18. The adjustive pval < 0.05 and |log2(Foldchange)| ≥ 2 were assigned as the statistically significant difference standard of DEG. The data reported in this paper were deposited in the OMIX, China National Center for Bioinformation/ Beijing Institute of Genomics, Chinese Academy of Sciences (https://ngdc.cncb.ac.cn/omix: accession no. OMIX001094) and NCBI Gene Expression Omnibus (GEO) repository (GSE GSE129030) 19. The Umap, feature, and violin plots were analyzed and presented using Seurat (4.1.0) 20. The dot plot and line chart are presented using ggplot2(3.35).

### T cell labeling with CellTrace CFSE or CellTrace Violet

To compare migration characteristics, *Ccr5*^-/-^ and WT DNT cells were labeled with 2.5 nM CellTrace CFSE (Thermo Fisher Scientific, USA) or 2 μM CellTrace Violet (CTV, Thermo Fisher Scientific), respectively, for 15 min at 37 °C in PBS without serum. The cells were washed twice with PBS containing 10% FBS to remove excess dye and adoptively transferred into ischemic stroke mice at a 1:1 ratio. A total of 5 million cells were injected intravenously into recipient mice.

### Real-time PCR

Total RNA was isolated from the infarct area using a TRIzol reagent (Sigma-Aldrich, USA) and reverse transcribed into cDNA using a SuperScript III RT kit (Invitrogen, CA, USA). Real-time PCR was performed by the ABI QuantStudio 3 (Applied Biosystems, USA) according to the manufacturer's instructions. Amplicon expression in each sample was normalized to Gapdh expression, and gene expression was subsequently quantified using the 2^-ΔΔCt^ method. The sequences of PCR primers are as follows: Ccl5, forward primer: 5′- GCTG CTTT GCCT ACCT CTCC -3′; reverse primer, 5′- TCGA GTGA CAAA CACG ACTGC-3′; Gapdh, forward primer: 5′- GGAG ATTG TTGC CATC AACGA -3′; reverse primer, 5′- GAAG ACAC CAGT AGAC TCCA CGACA -3′.

### Statistics

GraphPad Prism (version 8.0.2) was used to perform statistical tests and generate *P* values. All data points are included in the analysis. We used standard designation of *P* values throughout the figures (ns indicates not significant or *P* ≥ 0.05; *P < 0.05, **P < 0.01, ***P < 0.001, and ****P < 0.0001). Data are summarized as scatter plots with bars depicting means ± SEM. Details of the number of replicates are provided in the individual figure legends. A two-tailed unpaired Student's *t* test was used to compare unpaired two groups. A paired *t* test was used to compare *Ccr5*^-/-^ and WT DNT cell homing. We used two-way analysis of variance (ANOVA) to compare multiple groups, followed by Tukey's multiple comparisons. Further study data can be obtained by contacting the corresponding author.

## Results

### DNT cell therapy reduces infarct volume and promotes neurological functional recovery after ischemic stroke

**Figure 1:**
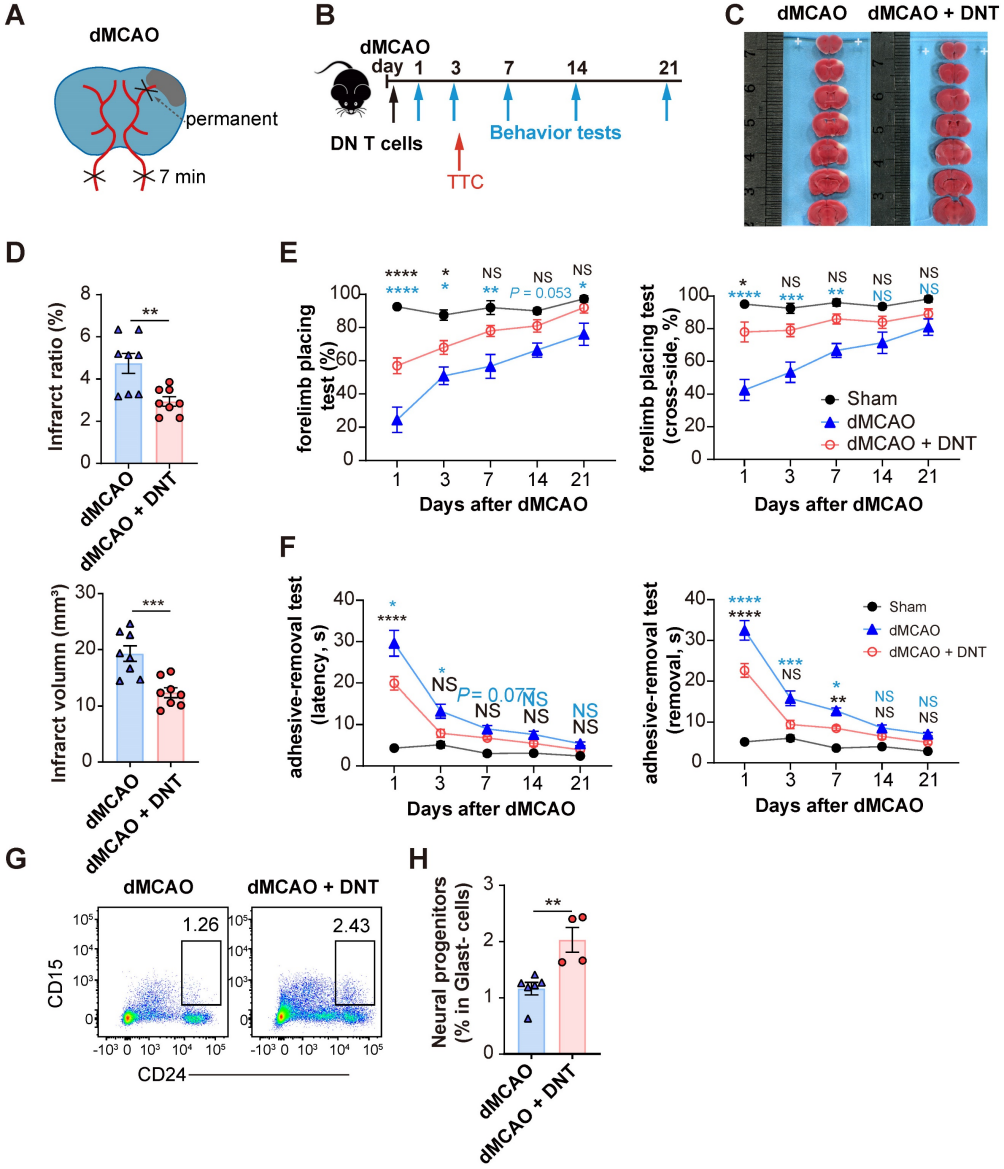
** DNT cell therapy promotes the functional recovery and neural repairment after ischemic stroke. (A)** Schematic strategy of the occlusion of the distal branches of the middle cerebral artery (dMCAO) for focal ischemic stroke model in mice. **(B)** Experimental design for DNT cell treatment and behavior assessment in ischemic stroke. **(C)** 2,3,5-Triphenyltetrazolium chloride (TTC) staining of brain slices 3 d after ischemic stroke surgery showing the infarct area in the barrel cortex (white). **(D)** The relative proportion and direct quantification of infarct volume by TTC staining. n = 8 mice/group. Two-tailed unpaired Student's t test. **(E and F)** Long-term sensorimotor functions after DNT cell treatment were assessed through (E) whisker-evoked forelimb placing and (F) adhesive-removal tests. Blue and black stars indicated the significant differences between dMCAO + DNT vs dMCAO and dMCAO + DNT vs sham group, respectively. n = 8-12 mice/group. Two-way analysis of variance (ANOVA) and Tukey.** (G and H)** (G) Representative flow plot and (H) quantification of CD24^hi^CD15^+^ neuronal progenitor in infarct area 21 d after ischemic stroke. n = 4-6 mice/group. Two-tailed unpaired Student's t test. ns indicates not significant or *P* ≥ 0.05; *P < 0.05, **P < 0.01, and ****P < 0.0001. Data are mean ± SEM.

C57BL/6J mice underwent ischemic stroke due to the dMCAO (Figure 1A) and were adoptively transferred with 5×10^6^ converted DNT cells or PBS without cells via tail vein injection within 30 min after ischemic stroke (Figure 1B). The infarct volume in DNT cell-treated mice was significantly reduced than that in control mice 3 d after dMCAO (Figure 1C-D). The adoptive transfer of DNT cells also potentiated long-term sensorimotor functions, as revealed by the increased success rate in the forelimb placing test (Figure 1E) and decreased time for the removal of adhesive tapes from the impaired paws in the adhesive-removal test (Figure 1F). To evaluate the effects of delayed DNT cell administration, DNT cells were administered 2 h after dMCAO. TTC staining also indicated a significant decrease in infarct volume in DNT-treated mice 3 d after dMCAO (Figure S2 AB). Moreover, the sensorimotor function was also improved after delayed DNT cell administration (Figure S2 C). CD15 and CD24^hi^ are considered as the surface markers of neuronal progenitors 21, 22. In this study, the proportion of Glast^-^CD24^hi^CD15^+^ neuronal progenitors in the infarct area was detected by flow cytometry. Our data indicated that DNT cell treatment significantly increases the proportion of neuronal progenitor in the chronic recovery phase (day 21) of ischemic stroke (Figure 1G-H). These results indicate that a single DNT cell treatment could achieve a neuroprotective effect and engender long-term neural recovery in dMCAO model mouse.

### DNT cell therapy affects neuroinflammation and neuroprotection of ischemic stroke

**Figure 2:**
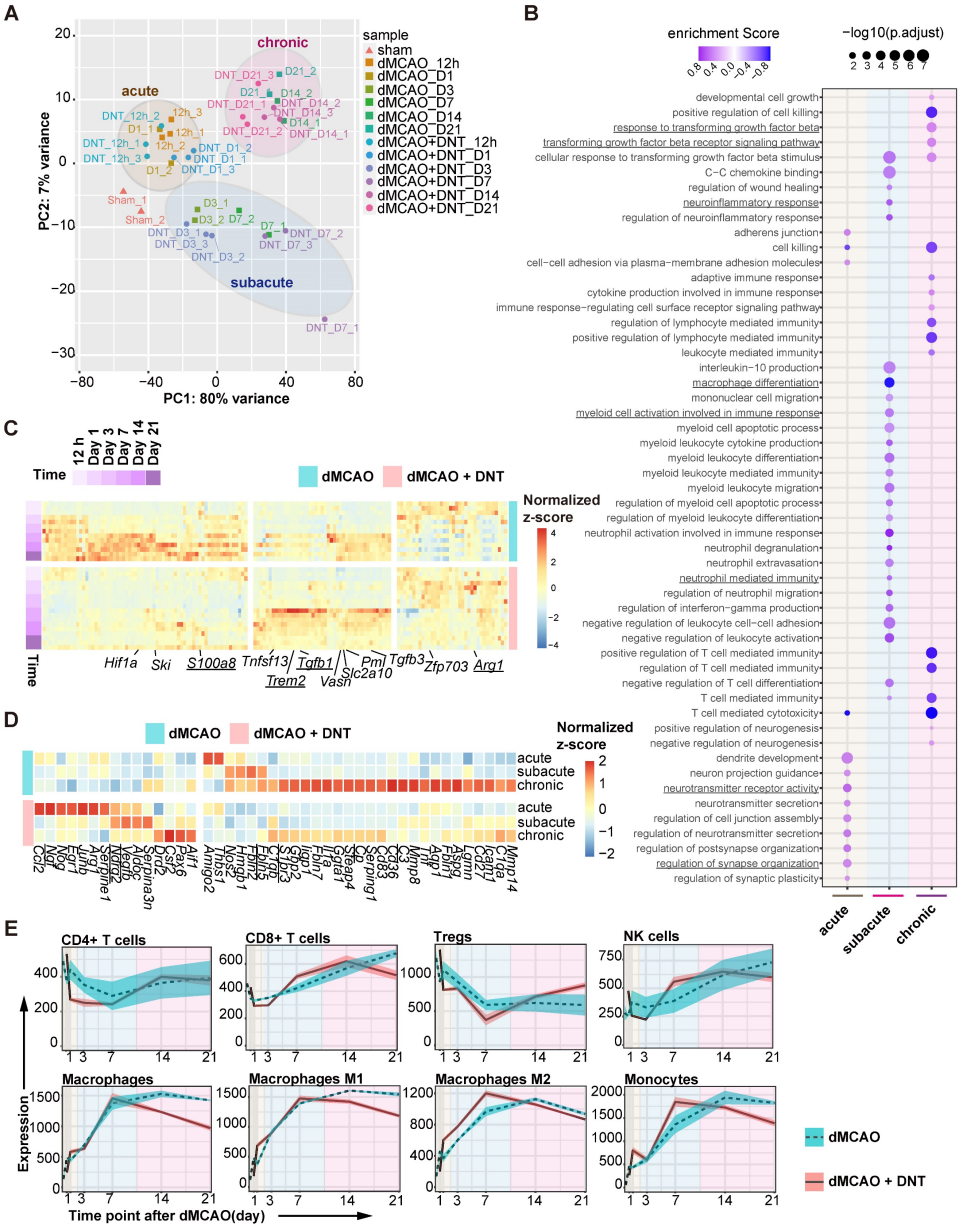
** DNT cell therapy regulates the immune homeostasis of ischemic tissue mainly during the subacute and chronic phases of ischemic stroke. (A)** Principal component analysis (PCA) of ischemic tissue bulk transcriptomes with or without DNT treatment 12 h to 21 d after occlusion of the distal branches of the middle cerebral artery (dMCAO). **(B)** Bubble map shows the changes of GO enrichment analysis of bulk RNA-seq after DNT treatment during the acute, subacute, and chronic phases in dMCAO mice ischemic areas. **(C)** Heatmap of immune-relevant gene expression by RNA sequencing after dMCAO with or without DNT treatment. **(D)** Heatmap of neurotoxic and neuroprotective gene expression in ischemic tissues in three phases by RNA-seq after dMCAO with or without DNT treatment. **(E)** XCell algorithm indicates immune cell variation by parenchyma bulk RNA sequencing.

To dissect the potential mechanism of DNT cell protection in ischemic stroke, bulk RNA sequencing on DNT-treated and untreated ischemic tissues was performed from 12 h to 21 d after dMCAO. PCA indicated that the samples clustered in groups with time-specific distinctions, suggesting three separate phases in dMCAO progress: acute (< 24 h), subacute (3-7 d) and chronic (14-21 d) (Figure 2A). Consistently, GSEA revealed that diverse pathways were significantly enriched in different phases after DNT cell treatment (Figure 2B). Synaptic plasticity and neurotransmitter signals, myeloid cell differentiation and function pathways, and immune cell and cytokine regulation pathways were enriched during the acute, subacute, and chronic phases, respectively. This analysis indicates that the residential influence of DNT cells transformed over time. Some neuroinflammation gene (*S100a8*, *Trem2*, *Tgfb1*, and *Arg1*) expression models were also conspicuously converted after DNT cell treatment in the ischemic tissues (Figure 2C). Moreover, DNT cell treatment significantly regulated neurotoxic (such as *Cp, Fbln5, S1pr3,* and* C3*) and neuroprotective (such as *Vegfb, Ndrg2,* and* Ngf)* gene expression during the acute, subacute, and chronic phases of dMCAO (Figure 2D). The online platform xCell was used to enumerate lymphocyte and myeloid cell subsets from RNA sequencing data. Compared to those of T cells and NK cells, the proportion and polarization of macrophages were dramatically regulated after DNT cell treatment in the subacute and chronic phases (Figure 2E). These bioinformatic analyses indicate that the immune regulation of DNT cells in infarct areas mainly functions during the subacute and chronic phases of ischemic stroke.

### DNT cells infiltrate into the ischemic brain tissue during the subacute and chronic phases of ischemic stroke

**Figure 3:**
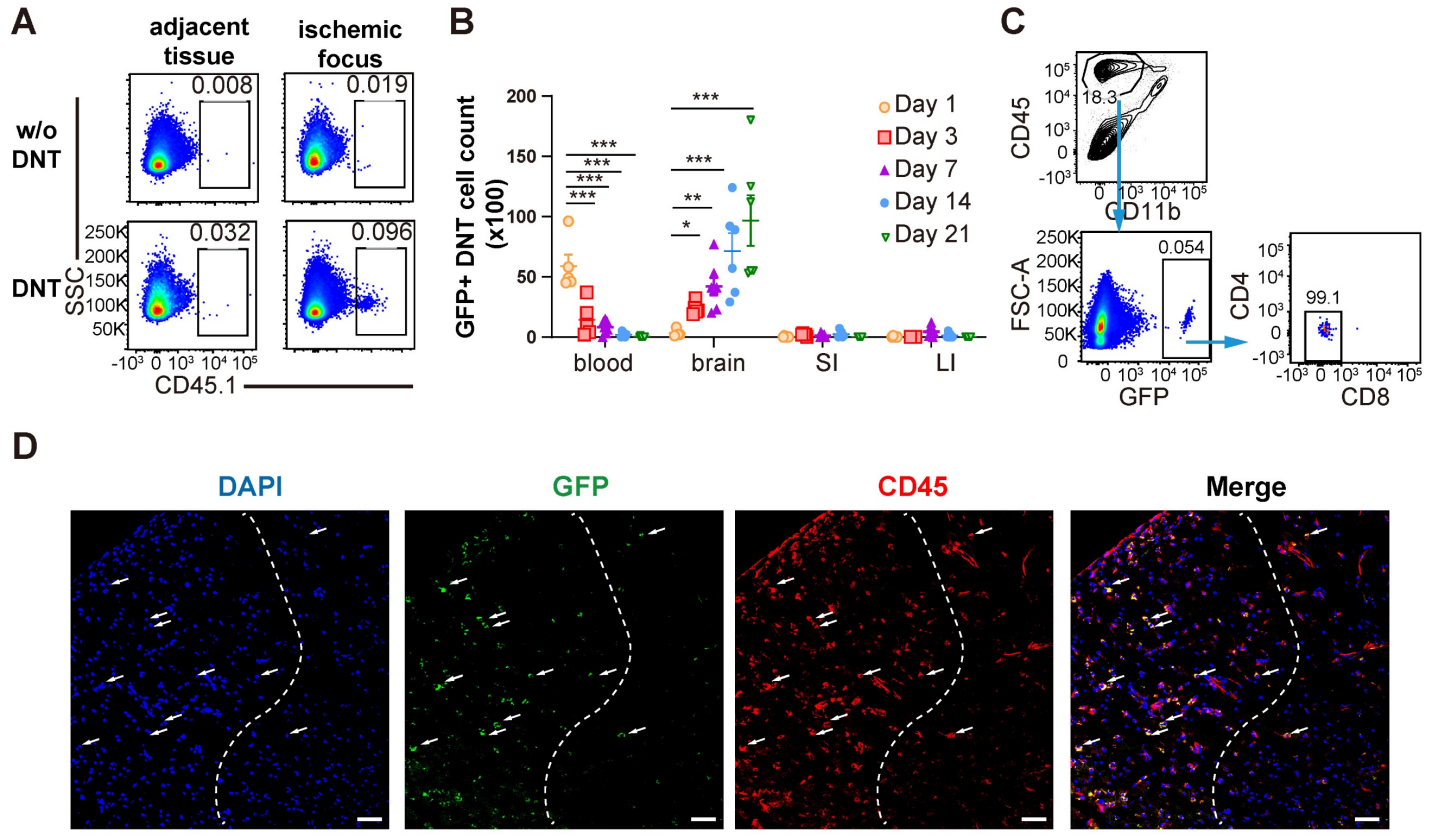
** DNT cells infiltrate into the tissue of the ischemic brain during the subacute and chronic phases of ischemic stroke. (A)** CD45.1^+^ DNT cells were adoptive transferred to mice intravenously after ischemic stroke. Flow cytometry analysis of CD45.1^+^ DNT cells in peripheral brain tissue and ischemic tissue at day 3. **(B-D)** GFP^+^ DNT cells were adoptive transferred to mice intravenously after ischemic stroke. (B) GFP^+^ cell counts in the peripheral blood, ischemic tissue of the brain, small intestine (SI), and large intestine (LI) were assessed using flow cytometry. n = 4 - 7 mice/group. Two-way analysis of variance (ANOVA). (C) CD4 and CD8 expression in GFP+ DNT cells 21 d after adoptive transfer. Blue arrows indicate the gating strategy. (D) Representative confocal images of GFP and CD45 double immunostaining in the infarct area obtained from occlusion of the distal branches of the middle cerebral artery (dMCAO) mouse treated with GFP DNT cells. The white dash line indicates the edge of ischemic area. Left side of the dash line: ischemic area, Right side of the dash line: normal area adjacent to ischemic area. Scale bar = 20 μm. White arrows denote CD45^+^ GFP^+^ DNT cells. *P < 0.05 and ****P < 0.0001. Data are mean ± SEM.

As a type of immune regulatory T cells, DNT cell-mediated immune regulation in infarct areas mainly during the subacute and chronic phases of dMCAO. To reveal the underlining mechanism, whether and when DNT cells migrated from the periphery to the brain of stroke mice were explored. DNT cells from CD45.1 mice were administered to dMCAO mice. CD45.1^+^ DNT cells accumulated in the ischemic tissue at 3 d, than the tissues adjacent to the ischemic area (Figure 3A). Recent studies have indicated that systemic inflammation occurred in peripheral blood and intestinal tissues 6, 23. Next, we compared the changes in DNT the cell counts in peripheral blood, brain infarct tissue, small intestine, and large intestine (Figure 3B). DNT cells stayed in the peripheral blood during the acute phase (24 h). While in contrast to the significant decrease in peripheral blood, the DNT cells in brain increased significantly since day 3 after dMCAO. Moreover, adoptively transferred DNT cells maintained the CD4^-^ and CD8^-^ phenotypes in ischemic tissue after 21 d (Figure 3C). Consistent with this, immunofluorescence indicated the migration of adoptively transferred GFP^+^ DNT cells in the infarct area on day 7 (Figure 3D). Taken together, these results suggest that the administered DNT cells circulate in the periphery during the acute phase and migrate to the ischemic area with a stable CD4^-^CD8^-^ phenotype during the subacute and chronic phases.

### DNT cells suppress peripheral Trem1^+^ myeloid cell differentiation during the acute phase

**Figure 4:**
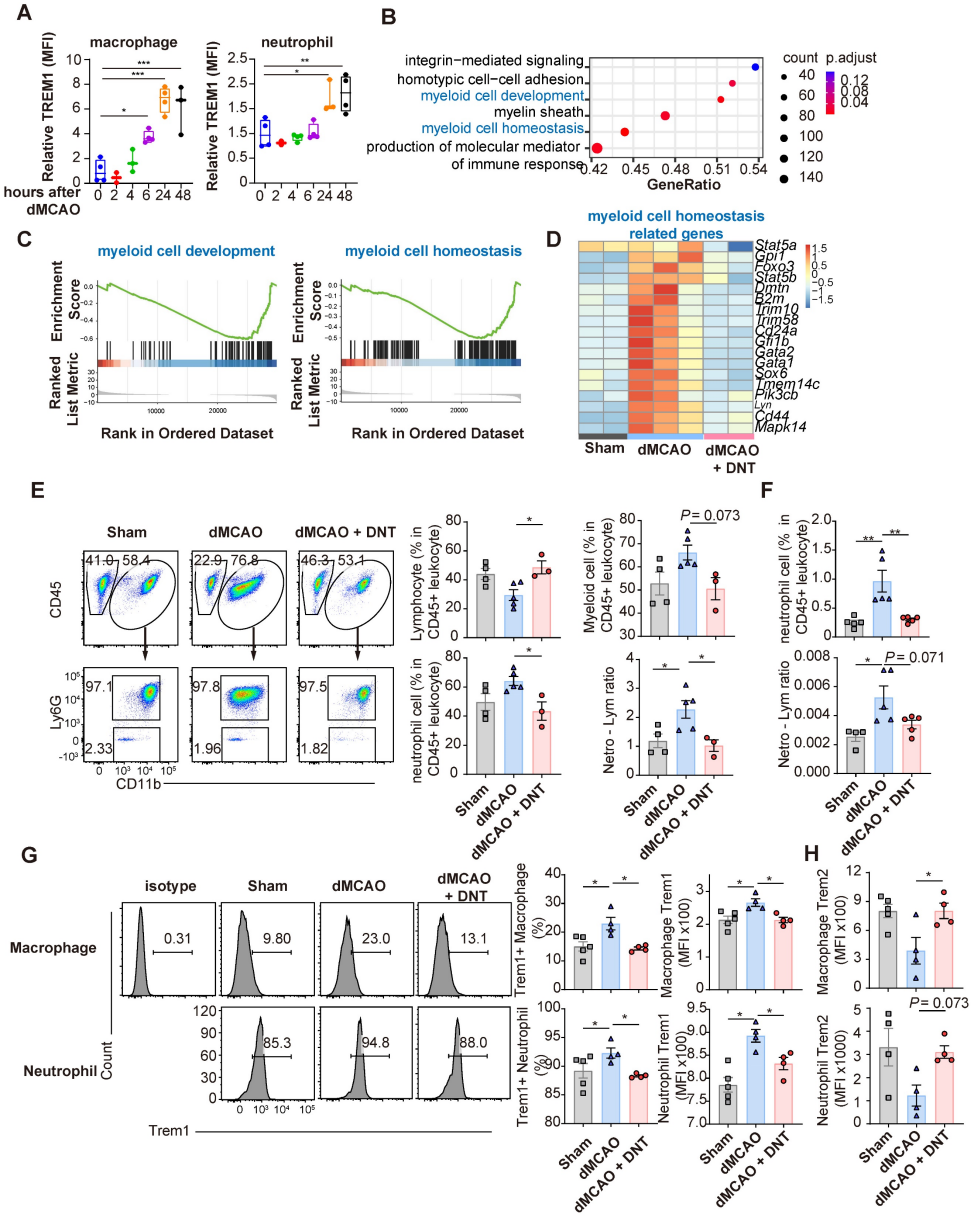
** DNT cells suppress the peripheral Trem1+ myeloid cell differentiation during the acute phase. (A)** Trem1 expression of peripheral macrophage different time points after occlusion of the distal branches of the middle cerebral artery (dMCAO). **(B)** GSEA analysis of gene expression in DNT cell-treated versus untreated PBMC.** (C)** Selected gene sets enriched in the DNT cell-treated PBMCs than those in the untreated PBMCs were plotted.** (D)** Heatmap of GSEA-enriched pathway genes in DNT cell-treated or untreated PBMC. **(E-H)** DNT cells were adoptive transferred to dMCAO mice, and peripheral immune status were assessed by flow cytometry after 24 h. **(E)** Proportions of CD45^+^ CD11b^-^ lymphocyte, CD45^+^ CD11b^+^ myeloid cells, CD45^+^ CD11b^+^ Ly6G^+^ neutrophil, and neutrophil-lymphocyte ratio in the peripheral blood after dMCAO. n = 3-5 mice/group. **(F)** Proportions of Ly6G^+^ neutrophil and neutrophil-lymphocyte ratio in spleen after dMCAO. n = 4-5 mice/group. **(G and H)** Flow cytometry depicts (G) Trem1 and (H) Trem2 expression in the peripheral neutrophils and macrophages. n = 4-5 mice/group. One-way analysis of variance (ANOVA) and Tukey's test. *P < 0.05, **P < 0.01, and ***P < 0.001. Data are mean ± SEM.

As shown above, infarct volume decreased on day 3 in mice after ischemic stroke. However, adoptively transferred DNT cells accumulated in the peripheral blood instead of the ischemic tissue 1 d after dMCAO. Moreover, the conversion of differentiation and Trem1 expression was not observed in the myeloid cells and microglia of the DNT cell-treated infarct tissues in the acute phase of dMCAO (Figure S3A-B). These data suggest that DNT cells may inhibit the peripheral inflammatory cells during the acute phase, limiting secondary immune injury in the brain. Ischemic stroke damages the blood-brain barrier (BBB) and releases DAMP, which intrigues innate immune cells after several hours. In line with previous reports, this study demonstrated that the expression of Trem1, a proinflammatory molecule, in peripheral macrophages and neutrophils was significantly upregulated during the acute phase of dMCAO (Figure 4A). To investigate the influence of DNT cells on peripheral immunity, the gene expression in PBMCs was explored through RNA sequencing 24 h after dMACO (Figure 4B-D). GSEA revealed that myeloid cell development and myeloid cell homeosis pathways were significantly enriched after DNT cell treatment (Figure 4B-C). The expression of critical transcriptional factors and effector molecules in myeloid cell differentiation was suppressed after DNT cell treatment (Figure 4D). Moreover, the neutrophil proportion and neutrophil-lymphocyte ratio, indicators of myeloid cell activation and poor stroke outcome in stroke 24, were significantly decreased in peripheral blood after DNT cell treatment (Figure 4E). Similar trends in neutrophil proportion and neutrophil-lymphocyte ratio were observed in splenic cells (Figure 4F). In addition, Trem1 expression in the macrophages and neutrophils of DNT cell-treated mice significantly decreased than that in those of untreated mice (Figure 4G). Consistently, Trem2, an anti-inflammatory molecule, was upregulated in peripheral neutrophils and macrophages after DNT cell treatment (Figure 4H). These data indicate that DNT cell treatment regulates proinflammatory myeloid cell differentiation in the acute peripheral phase. This effect may further influence the magnitude of damage or recovery in the subacute and chronic phases.

### DNT cells equilibrate the local immune balance by suppressing proinflammatory myeloid cells during the subacute stage

**Figure 5:**
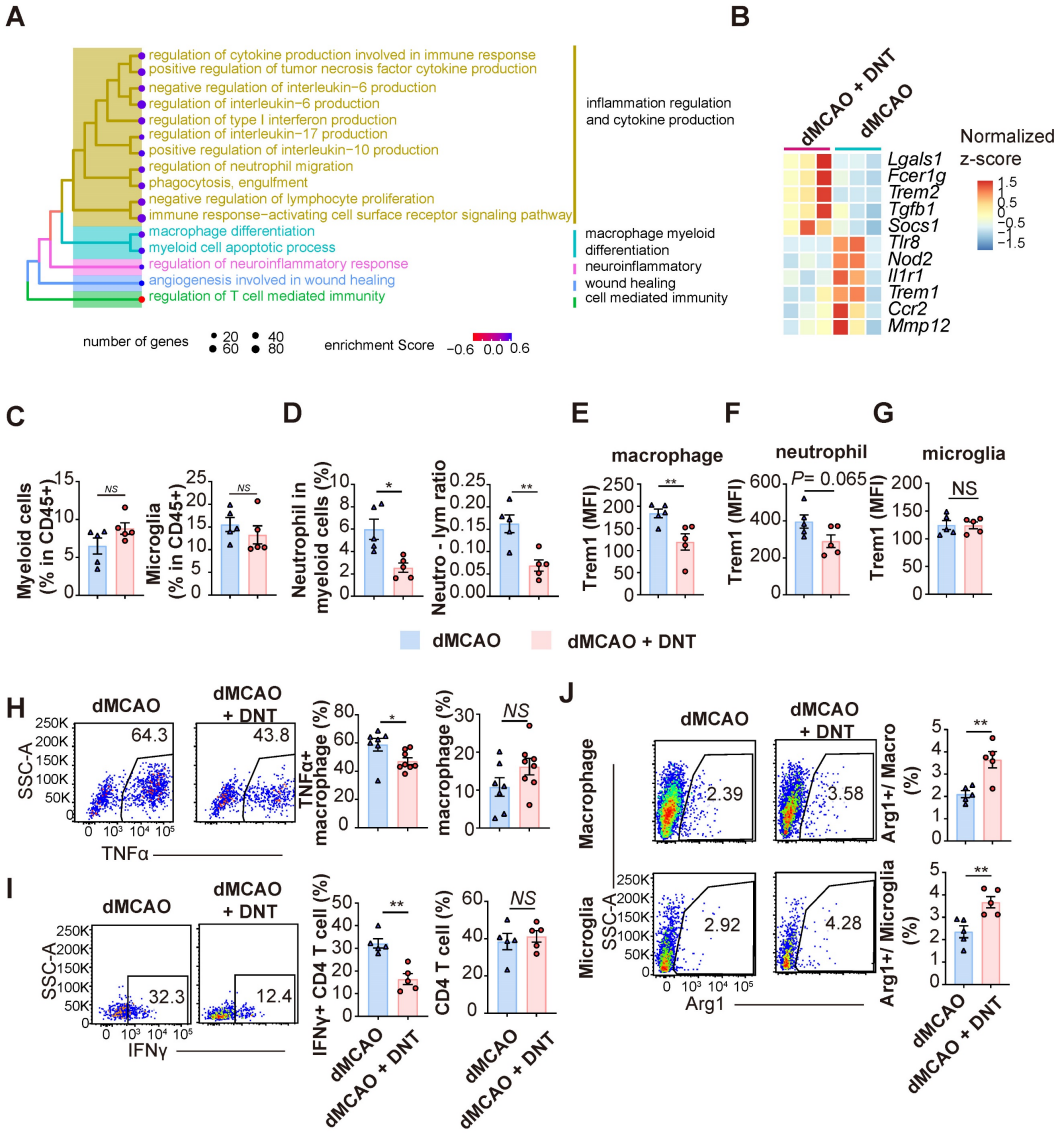
** DNT cells equilibrate the local immune balance by suppressing proinflammatory myeloid cells during the subacute stage. (A)** Treeplot shows GO enrichment terms' interrelation with ischemic stroke with or without DNT treatment using clusterProfiler (R package 4.2.2). (B) Heatmap of immune genes in DNT cell-treated or untreated lesion area. **(C-J)** DNT cells were adoptive transferred to occlusion of the distal branches of the middle cerebral artery (dMCAO) mice, and the immune status of ischemic tissue was assessed by flow cytometry after 7 d. **(C)** The proportions of CD45^+^ CD11b^+^ myeloid cells and CD45^mid^ CD11b^mid^ microglias in ischemic tissues. n = 5 mice/group. **(D)** CD45^+^ CD11b^+^ Ly6G^+^ neutrophils and neutrophil-lymphocyte ratio in ischemic tissues. n = 5 mice/group. **(E-G)** Flow cytometry depicts Trem1 expression in (E) macrophage, (F) neutrophil, and (G) microglia in ischemic tissues. n = 5 mice/group. **(H)** Flow cytometry depicts TNFα production in macrophage and the proportion of macrophage in immune cells in ischemic tissues. n = 7-8 mice/group.** (I)** Flow cytometry depicts IFNγ production in CD4^+^ T cells and the proportion of CD4^+^ T cells in T cells in ischemic tissues. n = 5 mice/group. **(J)** Flow cytometry depicts Arg1 expression in the macrophages and microglias. n = 5 mice/group. Two-tailed unpaired Student's t test. *P < 0.05 and **P < 0.01. Data are mean ± SEM.

To verify the effect of DNT cell treatment in the subacute phase, the transcriptomes of DNT cell-treated and untreated mouse ischemic tissues were compared 7 d after dMCAO. GSEA revealed that several inflammation regulation and myeloid cell differentiation pathways were enriched in the DNT cell-treated mice (Figure 5A). Several classical pro- and anti-inflammatory genes were downregulated and upregulated, respectively, after DNT cell treatment (Figure 5B). The immune environment in ischemic tissue was assessed using flow cytometry. The proportions of total CD45^+^CD11b^hi^ myeloid cells and CD45^mid^CD11b^mid^ microglia did not change significantly 7 d after DNT cell treatment (Figure 5C), whereas the proportion of neutrophils and neutrophil-lymphocyte ratio significantly decreased after DNT treatment (Figure 5D). Trem1 expression in macrophages markedly decreased after DNT treatment, which is consistent with the trend for neutrophil expression (Figure 5E-F). Trem1 expression did not change in the microglia, the resident myeloid cells present in the central nervous system (Figure 5G). TNFα is a critical proinflammatory cytokine produced by macrophages. With the unaffected proportion of total macrophage, TNFα production by macrophages in DNT-treated mice was also significantly reduced than that in control mice in the ischemic tissue (Figure 5H). Moreover, without regulating the proportion of CD4^+^ T cells, DNT treatment significantly mitigated the IFNγ secretion of CD4^+^ T cells (Figure 5I). In contrast, the expression of the anti-inflammatory macrophage marker, *Arg1*, increased in macrophages and microglia after DNT treatment (Figure 5J). These data suggest that the immune milieu of the ischemic tissue was equilibrated to an anti-inflammatory status with DNT treatment 7 d after dMCAO.

### DNT cells infiltrate into ischemic tissue via CCR5

**Figure 6:**
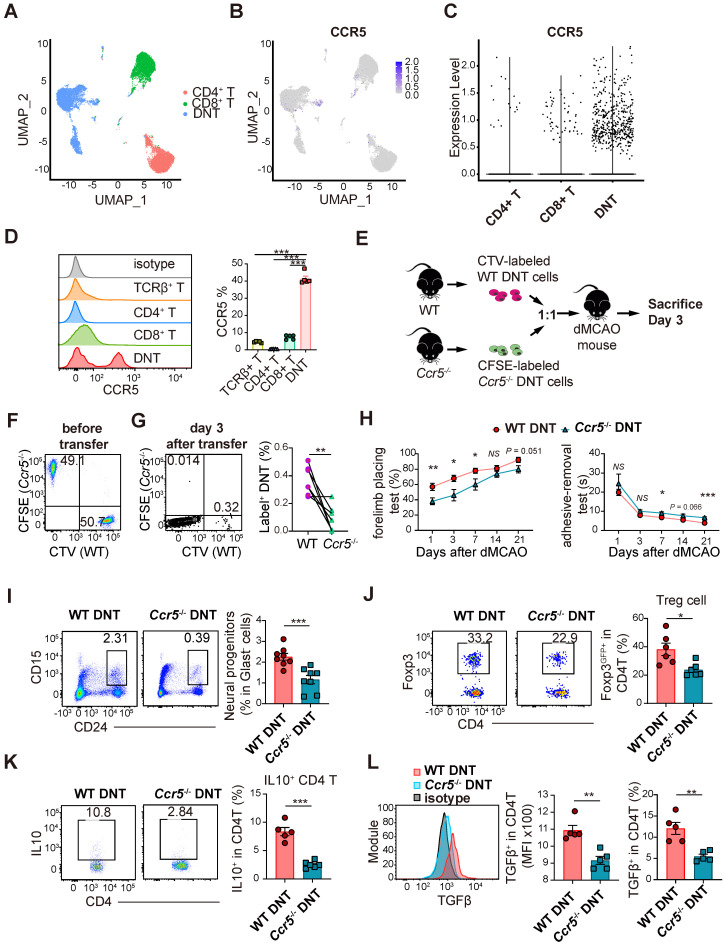
** DNT cells infiltrate into ischemic tissue via CCR5. (A)** UMAP showing the distribution of DNT, CD4, and CD8 sequencing using Seurat (R package,4.1.0). **(B)** Ccr5 expression in DNT, CD4 and CD8 cells by feature plot.** (C)** Violin plot showing Ccr5 expression in DNT, CD4, and CD8 cells. **(D)** CCR5 expression in splenic T cells was assessed by flow cytometry. One-way analysis of variance (ANOVA) and Tukey's test. n = 5 mice/group. **(E)** Experimental design for ischemic tissue homing of DNT cell assay. **(F)** Dot plots showing that CTV-labeled WT and CFSE-labeled *Ccr5*^-/-^ DNT cells were mixed as 1:1 before injection. **(G)** Dot plots showing migrated DNT cells 7 d after injection. Proportion of WT or *Ccr5*^-/-^ DNT cells (label+ DNT cells in total CD45+CD11b- cells) migrated to ischemic tissue. Paired t test, n = 7 mice/group. **(H)** Long-term sensorimotor functions after WT or *Ccr5*^-/-^ DNT cell treatment were assessed by whisker-evoked forelimb placing and adhesive-removal tests. n = 7-10 mice/group. Two-way analysis of variance (ANOVA) and Tukey's test. **(I and J)** Flow cytometry depicts the proportion of (I) CD24^hi^ CD15^+^ neuronal progenitors and (J) CD3^+^CD4^+^ Foxp3^GFP+^ Treg cells in the ischemic tissues 21 d after occlusion of the distal branches of the middle cerebral artery (dMCAO). n = 6-8 mice/group.** (K and L)** Flow cytometry depicts the (K) IL10 and (L) TGFβ production of CD4^+^ T cells in ischemic tissues 21 d after dMCAO. n = 4-6 mice/group. Two-tailed unpaired Student's *t* test. *P < 0.05, **P < 0.01 and ***P < 0.001. Data are mean ± SEM.

We have previously reported single-cell RNA sequencing of DNT, CD4^+^ T, and CD8^+^ T cells 19. Compared to that in CD4^+^ and CD8^+^ T cells, *Ccr5* expression was higher in DNT cells (Figure 6A-C). Moreover, flow cytometry data indicated that CCR5 expression was higher in DNT cells (Figure 6D). To investigate the role of CCR5 in DNT cell migration, WT and *Ccr5*^-/-^ DNT cells were stained with CTV and CFSE. WT and *Ccr5*^-/-^ DNT were simultaneously adoptively transferred into dMCAO mice at a 1:1 ratio (Figure 6E-F). Compared to WT DNT cells, migration of *Ccr5*^-/-^ DNT cells were mitigated significantly after 3 days of dMCAO (Figure 6G). Furthermore, to investigate the influence of *Ccr5* knockout on neural recovery effect of DNT cells, we treated dMCAO mice with WT and *Ccr5*^-/-^ DNT cells, respectively. As expected, compared to WT DNT cells, the recovery of long-term sensorimotor functions was weakened considerably after *Ccr5*^-/-^ DNT treatment (Figure 6H). Consistently, neuronal progenitor promotion weakened after *Ccr5*^-/-^ DNT treatment (Figure 6I).

To dissect the immunological status of the local tissue, the proportion and function of regulatory T cells were also determined by flow cytometry. The proportion of Foxp3^GFP+^ Treg cells in the ischemic tissues of *Ccr5*^-/-^ DNT-treated mice was less than that in WT DNT-treated mice (Figure 6J). Moreover, local CD4^+^ T cells in ischemic tissue produced less anti-inflammatory cytokines, IL-10 and TGFβ, after *Ccr5*^-/-^ DNT treatment (Figure 6K-L). These data show that DNT cells migrated to infarct tissue via CCR5, and neural repair and immune homeostasis maintenance were attenuated by *Ccr5*^-/-^ DNT treatment.

### DNT cells enhance Treg cell recruitment into tissue during the chronic phase

**Figure 7:**
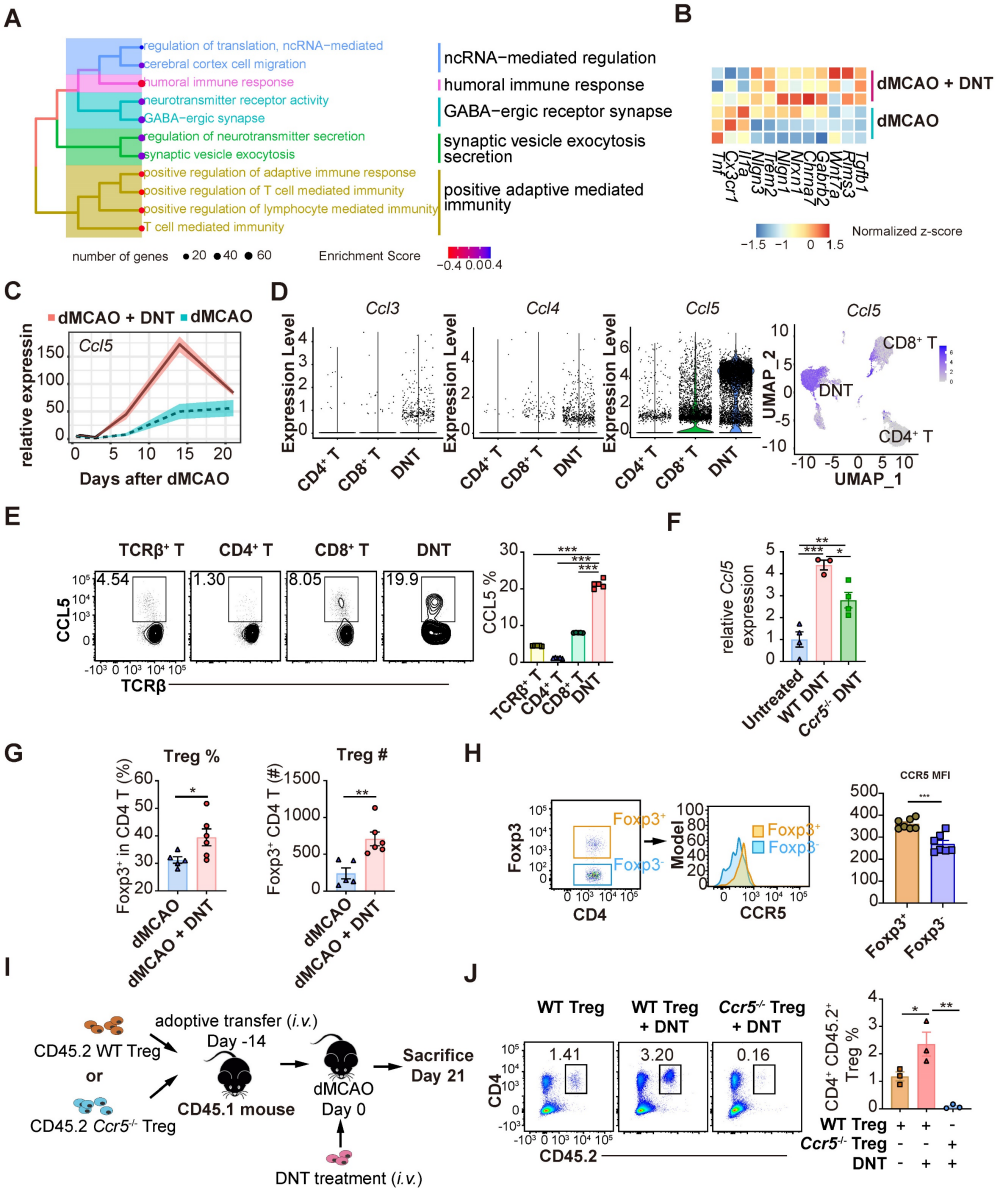
** DNT cells assist Treg cell recruitment into the ischemic tissue in the chronic phase. (A)** Treeplot shows GO enrichment terms' interrelation 21 d after ischemic stroke with or without DNT treatment using clusterProfiler (R package 4.2.2).** (B)** Heatmap of functional recovery genes in DNT cell-treated or untreated lesion areas 21 d after surgery. **(C)** Line chart showing Ccl5 expression in infarct area by parenchyma bulk RNA sequencing. **(D)** Violin plot showing Ccl3, Ccl4, and Ccl5 expression level in DNT, CD4^+^, and CD8^+^ cells and feature plot showing Ccl5 expression.** (E)** CCL5 production in splenic T cells was assessed by flow cytometry. n = 5 mice/group **(F)** Relative *Ccl5* transcription of infarct area after treatment was assessed by real-time PCR. **(G)** The proportion and number of Foxp3^ GFP+^ Treg cells in ischemic tissues 21 d after occlusion of the distal branches of the middle cerebral artery (dMCAO). n = 5-6 mice/group** (H)** CCR5 mean fluorescence intensity (MFI) quantification in Foxp3^GFP+^ and Foxp3^GFP-^ T cells in the ischemic tissue of stroke mouse were assessed by flow cytometry. n = 7 mice/group** (I-J)** WT or *Ccr5*^-/-^ CD45.2+ Treg cells were adoptively transferred intravenously to CD45.1 mouse 14 d before dMCAO. Ischemic stroke mice were treated by CD45.1^+^ DNT cells as above described and assessed 21 d after dMCAO. **(I)** Schematic representation of the experiment.** (J)** The proportion of CD4^+^CD45.2^+^ Treg cells in ischemic tissues 21 d after dMCAO. n = 3 mice/group. Two-tailed unpaired Student's *t* test or one-way analysis of variance (ANOVA). *P < 0.05, **P < 0.01, and ***P < 0.001. Data are mean ± SEM.

To explore the long-term impact of DNT cell treatment, the transcriptomes of DNT cell-treated and untreated mouse tissues were compared 21 d after dMCAO (Figure 7A-C). GSEA revealed that the neural recovery pathway and negative regulation of T cell immunity were enriched in DNT cell-treated mice (Figure 7A). Consistently, several anti-inflammatory and functional recovery-relevant genes were upregulated after DNT treatment (Figure 7B). Surprisingly, *Ccl5* expression in ischemic tissues increased by three-fold after DNT cell treatment (Figure 7C). Single-cell RNA-seq 19 indicated that *Ccl5* expression level in DNT cells is observably higher than that in other T cells (Figure 7D). Flow cytometry also verified a high proportion of CCL5 secretion in DNT cells (Figure 7E). Similar to the transcriptomic data, WT DNT cell treatment promoted local Ccl5 production 14 d after dMCAO. *Ccr5*^-/-^ DNT cell treatment also induced accumulated Ccl5 production, albeit at a lower level than WT DNT treatment (Figure 7F). These data suggest that besides DNT cells, other sources of Ccl5 in the infarct area also contributed to Treg migration to the brain during recovery. DNT cell treatment enhanced the proportion and number of Foxp3^GFP+^ Treg cells in ischemic tissues 21 d after dMCAO (Figure 7G). Moreover, compared to Foxp3^GFP-^ CD4^+^ T cells, Foxp3^GFP+^ Treg cells expressed a higher level of the CCL5 chemokine receptor CCR5 (Figure 7H). A previous study indicated that Treg cells dock at the injured vessel wall via CCR5 after an ischemic stroke 8. To investigate whether DNT cells assist the recruitment of CCR5^+^ Treg cells to the ischemic tissue, we adoptively transferred WT or *Ccr5*^-/-^ CD45.2^+^CD4^+^CD25^+^CD127^-^ Treg cells 14 d before dMCAO and DNT treatment (Figure 7I). This study showed that DNT cell treatment significantly promoted WT CD45.2^+^ Treg cell recruitment, whereas *Ccr5*^-/-^ CD45.2^+^ Treg cells failed to migrate to the ischemic tissue, even in DNT cell-treated stroke mice (Figure 7J). These results suggest that DNT cell therapy enhanced the recruitment of Treg cells during the chronic phase. The migration of Treg cell mainly depends on the expression of CCR5.

## Discussion

The approved therapies for acute ischemic stroke are thrombolytic therapy and endovascular thrombus removal 25. However, these therapies are associated with the increased risks of lethal hemorrhagic transformation and cerebral inflammation 26. Therefore, as we have learned more about inflammation after stroke in recent decades, therapeutic strategies that reduce systemic and cerebral inflammation may provide a clue for new therapies and improve the safety of current treatments.

Peripheral myeloid cells penetrate, accumulate, and affect cerebral injury during the acute phase of ischemic stroke 27. Trem1 expression is induced in peripheral myeloid cells within hours of cerebral ischemia 6. Trem1 and Trem2 expression in macrophages acts as an amplifier and brake, respectively, to the inflammatory response in insulin resistance, anti-tumor immunity, and liver injury 28, 29. These studies indicated the critical role of the Trem1 and Trem1/Trem2 balance in myeloid cells in secondary injury of stroke. Our data showed a significant decrease in peripheral Trem1^+^ myeloid cell differentiation after DNT cell treatment as early as 24 h after ischemic stroke. This early regulation of myeloid cell differentiation and polarization not only restricts the secondary inflammatory injury of infarct area, but may also contribute to the local immune homeostasis of ischemic areas in the subacute phase.

Along with the surface molecule expression that indicates cell polarization, proinflammatory and anti-inflammatory cytokines were also regulated after DNT cell treatment. As DAMPs are released from the damaged brain, innate immune cells are activated and quickly infiltrate the ischemic tissue through the BBB. Macrophages and neutrophils expressing proinflammatory cytokines play crucial roles in the acute and subacute phases of stroke 30. As a central proinflammatory cytokine produced by activated inflammatory cells, TNF-α promotes neuronal cell death 31, 32. Thus, various therapeutic strategies are aimed at suppressing TNF-α production to attenuate ischemic damage 33, 34. In this study, TNF-α production was significantly decreased in macrophages by DNT cell treatment 7 d after dMCAO, which is beneficial for further mitigation of neural damage. Arg1 is exclusively expressed in myeloid cells and essential for tissue repair during ischemic stroke 35. Moreover, Arg1 promotes efferocytosis in microglia and macrophages, which is instrumental in anti-inflammatory modality and restoration after stroke 36. Our previous study demonstrated that DNT cells selective suppressed the survival and TNFα production of proinflammatory macrophage via cell lysis 14. This study indicated increased Arg1 expression in macrophages in DNT-treated stroke mice. These data indicate a significant improvement in the immune milieu after DNT cell treatment.

As a vital anti-inflammatory lymphocyte, Treg cells accumulate 14 and 30 d after stroke and induce long-lasting immunological alteration 37. Emerging evidences indicate that brain regulatory T cells suppress astrogliosis and potentiate neurological recovery in ischemic stroke 7, 38, 39. Together with CCL5 upregulation in the endothelial cells of ischemic tissue, CCR5^+^ Treg cells dock to the injured epithelial cells 8. In this study, upregulated CCL5 expression in DNT cells facilitated Treg cell migration and maintenance at lesion sites. Recovery after stroke at day 21 might benefit from the synergistic effect of DNT and Treg cell accumulation and anti-inflammatory homeostasis.

A previous study showed the neuroinflammatory contribution of CD3^+^CD4^-^CD8^-^ DNT cells in middle cerebral artery occlusion (MCAO), another ischemic stroke model, which is more severe than most common stroke patients and the dMCAO model 40. dMCAO model is suitable for assessing long-term treatment effect and surveying neuroinflammation 41. As TCRγδ^+^ T cells and NKT cells were not excluded from CD3^+^CD4^-^CD8^-^ DNT cells, the proinflammatory outcome of CD3^+^CD4^-^CD8^-^ DNT cells is a combined effect of TCR αβ DNT cells, TCR γδ T cells, and NKT cells. TCR γδ T cells account for most CD3^+^CD4^-^CD8^-^ DNT cells and increase infarct volume by IL-17 secretion 42. Moreover, the effects of NKT cells on cerebral stroke severity are still remain controversial 43. Our recent study also demonstrated that, in contrast to TCRαβ^+^ DNT cells, TCRγδ^+^ DNT cells exert proinflammatory effects in the inflammatory tissue of non-alcoholic steatohepatitis 44. Due to these contradictory characteristics of DNT cell subsets, we excluded TCRγδ+ DNT and NKT cells in this study. Thus, this study complements the previous study by verifying the critical anti-inflammatory role of TCRαβ^+^NK1.1^-^ DNT, a subset of CD3^+^CD4^-^CD8^-^ DNT cells.

In conclusion, a single transfer of DNT cells showed promising immunomodulatory and neuroprotective properties following ischemic stroke in mice. First, DNT cells inhibited brain damage-triggered peripheral inflammation and restricted secondary brain injury during the acute phase of ischemic stroke. Second, DNT cells migrate to infarct areas in the subacute phase of ischemic stroke, restore immune homeostasis, and protect the brain from further damage. Finally, DNT cells enhance Treg cell recruitment and facilitate functional recovery after an ischemic stroke. This study provides a potential application for DNT cell-based therapy for ischemic stroke.

## Supplementary Material

Supplementary figures.

## Abbreviations

DNT: double negative T
dMCAO: occlusion of the distal branches of the middle cerebral artery
CCR5: C-C Chemokine Receptor 5
CCL5: C-C Motif Chemokine Ligand 5
DAMPs: damage-associated molecular patterns
Treg cells: regulatory T cells
Trem1: triggering receptor expressed on myeloid cells-1
TTC: 2,3,5-Triphenyl tetrazolium chloride
