# Supplementary material for: TCRαβ+NK1.1-CD4-CD8- double-negative T cells inhibit central and peripheral inflammation and ameliorate ischemic stroke in mice
Source: Theranostics. 2023 Jan 10;13(3):896–909. doi: 10.7150/thno.80307 (PMC9925325; doi:10.7150/thno.80307)
Supplement: Supplementary file 1 — Supplementary figures. [file thnov13p0896s1.pdf]

# Supplementary materials

# Figure S1

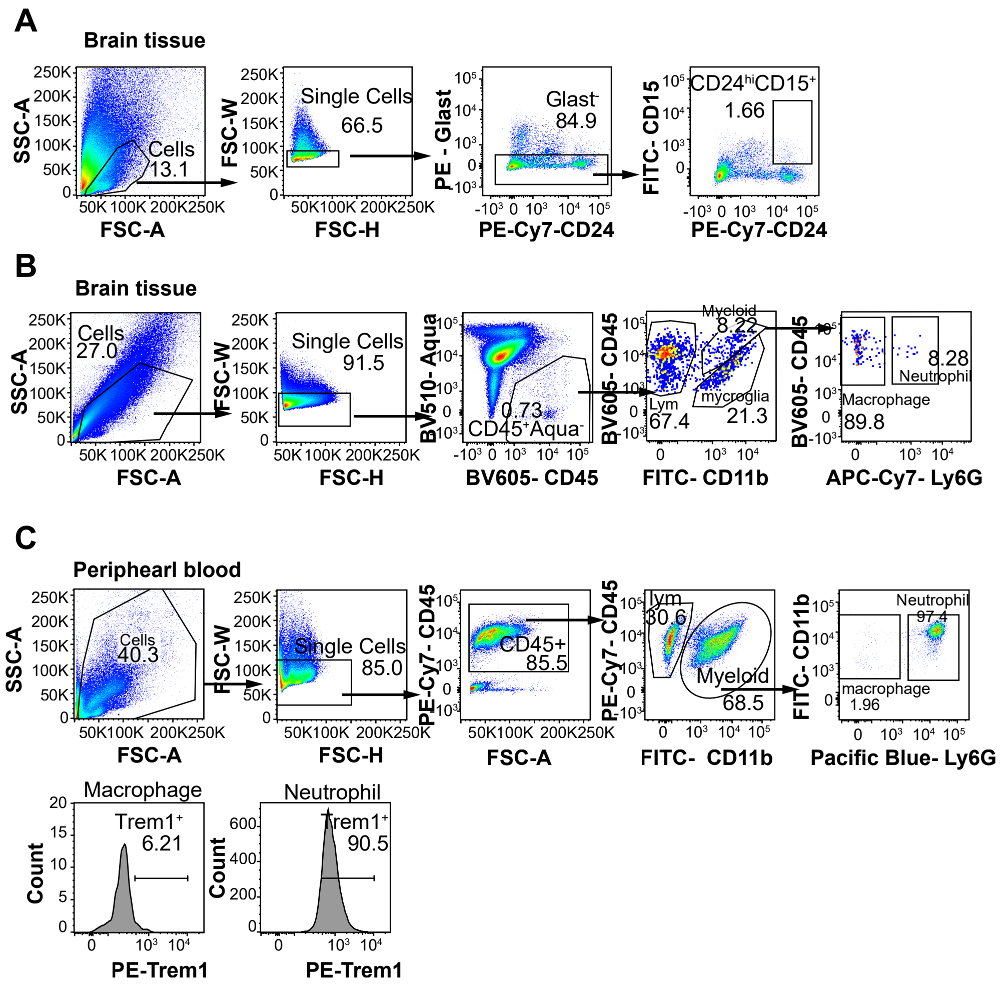


**Figure S1. Gating strategies of neural progenitors and immune cells in mouse brain tissues and peripheral blood.**

(**A**) Gating strategy to identify neural progenitors (Glast$^-$ CD24$^{hi}$ CD15$^+$) in infarct area of mouse brain. (**B**) Gating strategy to identify lymphocytes (CD45+ CD11b-), microglia (CD45$^+$ CD11b$^{low}$), macrophage (CD45$^+$ CD11b$^+$ Ly6G$^-$) and neutrophils (CD45$^+$ CD11b$^+$ Ly6G$^+$) in infarct area of mouse brain. (**C**) Gating strategy to identify lymphocytes, myeloid cells (CD45$^+$ CD11b$^+$), macrophage (CD45$^+$ CD11b$^+$ Ly6G$^-$) and neutrophils (CD45$^+$ CD11b$^+$ Ly6G$^+$) and Trem1 expression of macrophage and neutrophil in mouse peripheral blood.

# Figure S2

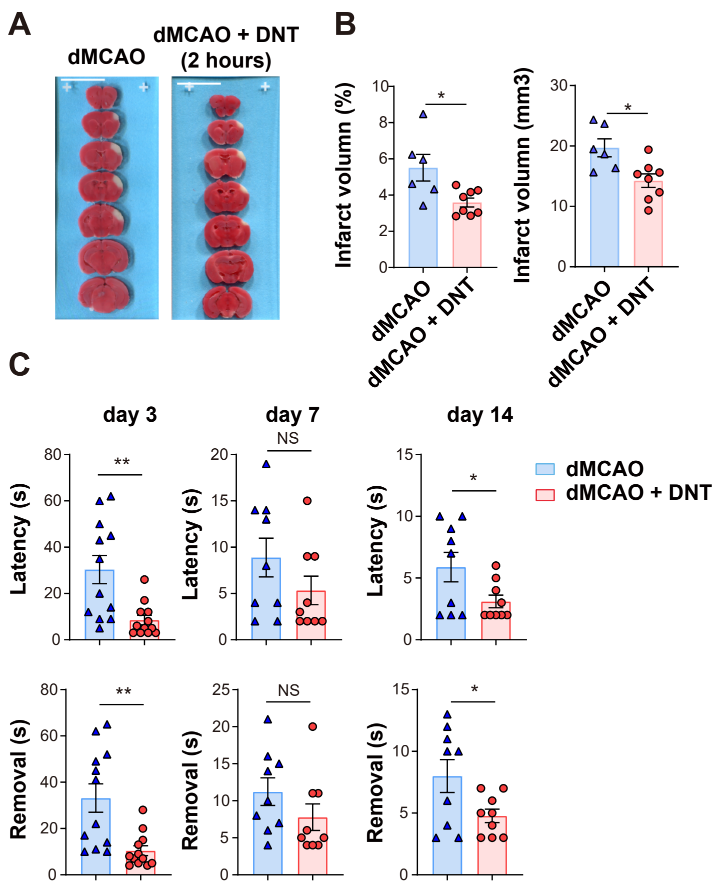


**Figure S2. Double-negative T (DNT) cell treatment 2 h after ischemic stroke promotes the recovery.**

DNT cells were administered 2 h after occlusion of the distal branches of the middle cerebral artery (dMCAO) and 2,3,5-triphenyltetrazolium chloride (TTC) staining was performed 3 d after dMCAO. (**A**) TTC staining of brain slices 3 d after ischemic stroke showing the infarct area in the cortex (white). (**B**) Relative proportion and direct quantification of the infarct volume by TTC staining. n = 6-8 mice/group. (**C**) Sensorimotor functions after DNT cell treatment were assessed through adhesive-removal tests at day 3, 7, and 14 after dMCAO. Two-tailed unpaired Student's *t* test. *$P < 0.05$. Data are mean ± SEM.

# Figure S3

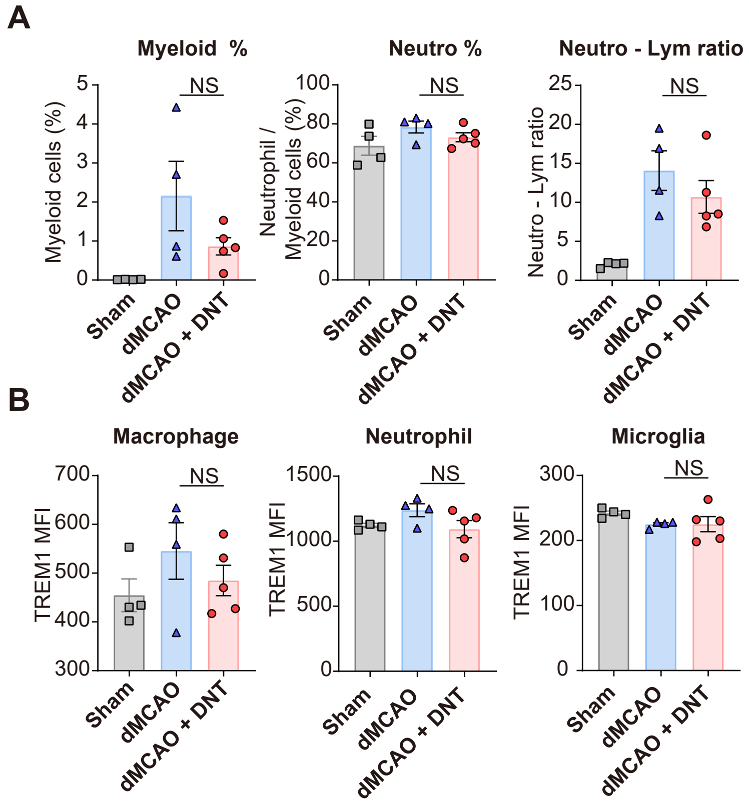


**Figure S3. Double-negative T (DNT) cells could not significantly impact the local myeloid cell differentiation 24 h after occlusion of the distal branches of the middle cerebral artery (dMCAO).**

(**A**) Proportions of $CD45^{+}CD11b^{hi}$ myeloid cell, $CD45^{+}CD11b^{hi}$ $Ly6G^{hi}$ neutrophil, and neutrophil–lymphocyte ratio were detected by flow cytometry. (**B**) Trem1 MFI of myeloid cell were detected by flow cytometry. n = 4–5 mice/group. Analysis of variance (ANOVA). NS indicates not significant. Data are mean ± SEM.
